# Clear Cell Papillary Renal Cell Carcinoma Shares Distinct Molecular Characteristics and may be Significantly Associated With Higher Risk of Developing Second Primary Malignancy

**DOI:** 10.3389/pore.2021.1609809

**Published:** 2021-08-27

**Authors:** Xi Tian, Wen-Hao Xu, Jun-Long Wu, Hua-Lei Gan, Hong-Kai Wang, Wei-Jie Gu, Yuan-Yuan Qu, Hai-Liang Zhang, Ding-Wei Ye

**Affiliations:** ^1^ Department of Urology, Fudan University Shanghai Cancer Center, Shanghai, China; ^2^ Department of Oncology, Shanghai Medical College, Fudan University, Shanghai, China; ^3^ Department of Pathology, Fudan University Shanghai Cancer Center, Shanghai, China

**Keywords:** somatic mutation, clear cell papillary renal cell carcinoma, germline mutation, fanconi anemia pathway, second primary malignancy

## Abstract

Traditionally, clear cell papillary renal cell carcinoma (ccpRCC) was considered to share similar molecular and histological characteristics with clear cell renal cell carcinoma (ccRCC) and papillary renal cell carcinoma (pRCC). Here we aimed to identify somatic and germline variants of ccpRCC. For this purpose, we conducted whole-exome sequencing to detect somatic variants in the tissues of 18 patients with pathologically confirmed ccpRCC, who underwent surgical treatment at Fudan University Shanghai Cancer Center. Targeted sequencing was conducted to detect germline variants in paired tumor or normal tissues or blood. Somatic and germline variants of ccRCC and Renal cell carcinoma included in The Cancer Genome Atlas data and other published data were analyzed as well. The molecular profiles of ccpRCC, ccRCC and pRCC were compared. Among the 387 somatic variants identified, *TCEB1* (3/18) and *VHL* (3/18) variants occurred at the highest frequencies. Germline mutation detection showed that nine variants associated with Fanconi anemia (VAFAs) pathway (*FANCA*, 6/18; *FANCI*, 3/18) were identified in 18 ccpRCC patients. Among ccpRCC patients with VAFAs, five out of eight patients had second primary malignancy or family history of cancer. Somatic variants characteristics may distinguish ccpRCC from ccRCC or pRCC and germline VAFAs may be a molecular characterization of ccpRCC. Compared with ccRCC or pRCC, ccpRCC patients may be significantly correlated with higher risk of developing second primary malignancy.

## Introduction

Renal cell carcinoma (RCC) is the third most common malignant tumor of the genitourinary system. In 2019, 431,288 renal tumors were newly diagnosed and179,368 patients were dead of kidney cancer[1]. Clear cell renal cell carcinoma (ccRCC) accounts for approximately 70% of adult RCC[2], and papillary renal cell carcinoma (pRCC) is the most common non-clear cell RCC, accounting for 10–15% of RCC[3]. Other subtypes of RCC include chromophobe RCC and collecting duct carcinomas. These pathological types differ in prognosis and may require different treatment strategies; therefore, they must be definitively diagnosed. Unfortunately, it may be difficult to distinguish between subtypes using histological and immunohistochemical analyses[2].

Clear cell papillary RCC (ccpRCC), initially described in 2006 in association with end-stage renal disease, was classified in 2016 as a discrete renal neoplasm by the World Health Organization. ccpRCC is characterized by unique as well as common morphological and molecular characteristics compared with those of ccRCC and pRCC[3]. In particular, ccpRCC, which rarely occurs, typically comprises a mixture of cystic and papillary components circumscribed with a fibrous capsule[4]. Furthermore, ccpRCC tumor cells express characteristic immunohistochemical (IHC) markers, and most ccpRCC cells express cytokeratin (CK)-7 and carbonic anhydrase (CA)-IX, but typically not membrane metalloendopeptidase (CD10)[5-7]. Evidence indicates that ccpRCCs are indolent tumors with low malignant potential[8, 9]. In contrast, morphological, immunohistochemical, and molecular genetic analyses indicate that at least one case of a metastatic renal tumor may represent ccpRCC[10]. Thus, the genotypic and phenotypic properties of ccpRCC may be more complex than previously demonstrated.

To pursue this possibility, here we conducted next-generation sequencing (NGS), which has profoundly contributed to our understanding of oncogenesis [11]. For example, a targeted NGS analysis of 50 genes in ccpRCC cells identified somatic variants in the *MET* proto-oncogene, which is associated with the epithelial-to-mesenchymal transition, which may play a key role in ccpRCC[12]. Another NGS study of 90 genes (combined with analysis of single-nucleotide polymorphisms) of ccpRCC cells revealed significant genotypic heterogeneity and a molecular profile similar to that of ccRCC[3]. Our analysis here aims to find potential characteristic germline and somatic variants of ccpRCC.

To further characterize the molecular genetic basis for these findings, we conducted whole-exome sequencing as well as targeted sequencing of 63 genes, selected by the National Comprehensive Cancer Network (NCCN) guidelines (https://www.nccn.org/guidelines/guidelines-detail?category = 1&id = 1440), to identify somatic and germline variants. Moreover, we analyzed The Cancer Genome Atlas (TCGA) data to further compare the molecular genetic features of ccpRCC, ccRCC, and pRCC to develop specific diagnostic tests and tumor markers. To our knowledge, this study employed the largest sample size among published studies using WES to identify somatic variants in patients with ccpRCC. Furthermore, the present study is the first to our knowledge to search for germline variants in such patients.

## Materials and Methods

### Clear Cell Papillary Renal Cell Carcinoma Samples

This study included 18 patients (age-range, 25–77 years) with histopathologically confirmed ccpRCC who underwent surgery at FUSCC between 2010 and 2019. Tumor specimens were obtained with patients’ informed consent. The 4-μm-thick sections from the formalin-fixed paraffin-embedded representative ccpRCC tissue blocks were deparaffinized. Antigen retrieval was performed with 10 mM citrate buffer solution (pH 6.0) in a pressure cooker (20 psi for 10 min). Endogenous peroxidase was quenched in 3% hydrogen peroxide for 15 min at 37°C and nonspecific binding was blocked with 10% normal goat serum for 1 h at room temperature. Sections were then incubated with the primary antibody at 4°C overnight. Chromogenic detection was carried out and DAB reagents were provided in the Envision detection kit (Dako). Tissue sections were counterstained with Meyer’s Haematoxylin (Thermo Fisher Scientific, Waltham, MA, United States). Omission of the primary antibody with phosphate-buffered saline served as a negative control. Primary antibodies used in this research were listed in Table 1. The diagnosis was established following standard morphological and immunohistochemical (IHC) criteria as follows: IHC detection of CK7 and CAIX and predominantly undetectable IHC detection of CD10. After reviewing hematoxylin and eosin-stained slides and IHC data, formalin-fixed paraffin-embedded tissue blocks were selected from each case for molecular analysis. Patients’ clinicopathological characteristics are listed in Table 2.

**TABLE 1:** Primary antibody used in this study.

Markers	Antibody names	Provider	Catlog
CD117	Anti-c-Kit antibody	abcam	ab32363
CD10	Anti-CD10 antibody	abcam	ab256494
TFE3	Anti-TFE3 antibody	abcam	ab179804
Ki67	Anti-Ki67 antibody	abcam	ab15580
CK7	Anti-Cytokeratin 7 antibody	abcam	ab68459
CAIX	Anti-Carbonic anhydrase 9 antibody	bcam	ab243660
Vimentin	Anti-Vimentin antibody	abcam	ab92547

**TABLE 2:** Clinicopathological characteristics of 18 ccpRCC patients (Fudan University Shanghai Cancer Center cohort).

case	Age	Gender	Stage	Fuhrman grade	Family history of cancer (7/18, 38.9%)	Second primary cancer (5/18, 27.8%)	Follow-up (month)
1	25	M	I	2	NA	NA	120
2	51	M	I	2	NA	GC	70
3	59	F	I	2	NA	EC	66
4	57	M	I	2	Brother and father with CRC	NA	56
5	63	M	I	2	NA	GC	54
6	72	M	I	2	Brother with HCC, sister with BC	PC	48
7	72	M	I	2	Mother with GBC	NA	47
8	77	M	I	2	One brother with HCC and one with PC	NA	44
9	63	M	I	2	NA	NA	43
10	40	M	I	2	NA	NA	41
11	66	M	I	2	NA	NA	29
12	54	M	I	2	Father with ESCC	NA	29
13	45	F	I	2	NA	NA	28
14	66	M	I	3	Father with HCC	GC	27
15	40	F	I	2	Father with TC	NA	23
16	35	M	I	2	NA	NA	17
17	62	M	I	3	NA	NA	12
18	54	M	I	2	NA	NA	11

Abbreviations: BC, breast cancer; ccpRCC, clear cell papillary Renal Cell Carcinoma; CRC, colorectal cancer; EC, Endometrial cancer; ESCC, esophageal squamous cell carcinoma; F, female; GBC, gallbladder cancer; GC, Gastric cancer; HCC, hepatocellular carcinoma; L, left; M, male; NA, not applicable; R, right; PC, prostate cancer; TC, thyroid cancer.

### Next Generation of Sequencing Analysis

WES was used to detect somatic variants, and targeted sequencing of 63 genes, selected per the NCCN guidelines (Table 3), was applied to detect germline variants in ccpRCC samples and paired normal tissues or blood (performed by Origi-Med (Shanghai, China). DNA was extracted using a QIAamp DNeasy blood and tissue kit (Qiagen, Valencia, CA, United States). BWA-MEM aligner was used to align raw reads with the reference genome (hg19), and Assembly Based ReAligner[13] was used for further remapping and recalibration. Quality control included correcting sequencing errors, coverage distribution, and insert size estimates. Picard software was used to remove polymerase chain reaction duplications. BAM files were analyzed for variants and genomic alterations (amplification, deletions). An SNP cut-off rate of 0.95, the minimum allele frequency of 0.05 were implemented in the analysis and functional annotation was performed by ANNOVAR.

**TABLE 3:** 63 genes for germline testing (NCCN guideline-recommended).

63 genes for germline testing		
APC	FH	POLE
ATM	FLCN	PRSS1
AXIN2	GALNT12	PTEN
BAP1	GREM1	RAD50
BARD1	HOXB13	RAD51C
BLM	MEN1	RAD51D
BMPR1A	MET	RB1
BRCA1	MITF	RET
BRCA2	MLH1	RHBDF2
BRIP1	MRE11	SDHA
CDH1	MSH2	SDHB
CDK12	MSH3	SDHC
CFTR	MSH6	SDHD
CHEK2	MUTYH	SMAD4
EGFR	NBN	SPINK1
EPCAM	NF1	STK11
FANCA	NF2	TP53
FANCC	NTHL1	TSC1
FANCD2	PALB2	TSC2
FANCG	PMS2	VHL
FANCI	POLD1	XRCC2

### Comparison of Genotypes of Clear Cell Papillary Renal Cell Carcinoma, Clear Cell Renal Cell Carcinoma and Papillary Renal Cell Carcinoma

Somatic variants of ccRCC and pRCC included in TCGA data were obtained from the UCSC genome browser (https://xenabrowser.net/datapages/). Germline variants of ccRCC and pRCC were obtained from a previous study[14]. The 20 genes with the highest frequencies of somatic and germline variants were displayed using the ComplexHeatmap package[15]. Somatic variants characteristic of ccRCC included those of *PBRM1*, *VHL*, *BAP1,* and *SETD2*, which are associated with the monoallelic loss of chromosome 3p, as well as genes encoding components of the PI3K signaling pathway (*PIK3CA*, *mTOR*, *PTEN*)[16]. Somatic variants of pRCC include variants in *MET,* hippo pathway-associated genes (*WWC1*, *SAV1*, *NF2*), chromatin modification-associated genes (*SETD2*, *KDM4B*, *KDM6A*), and NRF2 pathway-associated genes (*CUL3*, *KEAP1*, *NFE2L2*)[17]. We compared the frequencies of somatic variants in these genes among ccpRCC, ccRCC, and pRCC.

Germline variants in *MET* and *FH* are strongly associated with pRCC, and germline variants in *BAP1* and *VHL* contribute to the oncogenesis of ccRCC[14]. In the present study, we detected a high percentage of the Fanconi anemia (FA) mutation in patients with ccpRCC. Thus, pathogenic variants and variants of uncertain significance (VUSes) of *MET*, *FH*, *BAP1*, *VHL,* and FA-associated genes (*FANCM*, *FANCI*, *FANCC*, *FANCA*) were included in the analyses of germline variants. The clinical significance of a mutation was obtained from ClinVar (httpfs://www.ncbi.nlm.nih.gov/clinvar/). The functional prediction of VUSs was performed using PolyPhen-2 (http://genetics.bwh.harvard.edu/pph2/).

## Results

### Clear Cell Papillary Renal Cell Carcinoma Patients Have Unusually High Rates of a Family History of Cancer and Second Primary Cancers

The 18 patients included in the present study were diagnosed with stage-I ccpRCC. We were surprised to find a relatively high rate of family history of cancer (7/18, 38.9%) and second primary cancers (5/18, 27.8%) in ccpRCC. Although a previous study[18] indicated higher rates of family history of cancer in renal cell carcinoma patients (62.96%), it was still unexpected to find nearly 40% rates of family history of cancer in this neoplasm which is usually considered benign. Studies have reported incidences of second primary malignancy in renal cell carcinoma patients to range from 10%[19], 13%[20] and 16%[21], while patients with ccpRCC have a relatively higher incidence of second primary cancers. Thus, we hypothesized that patients with ccpRCC may harbor characteristic germline variants. All patients were alive at the last follow-up (median follow-up, 42.5 months; range, 11–120 months).

### Distinct Histological and Immunohistochemical Features of Clear Cell Papillary Renal Cell Carcinoma

**FIGURE 1:**
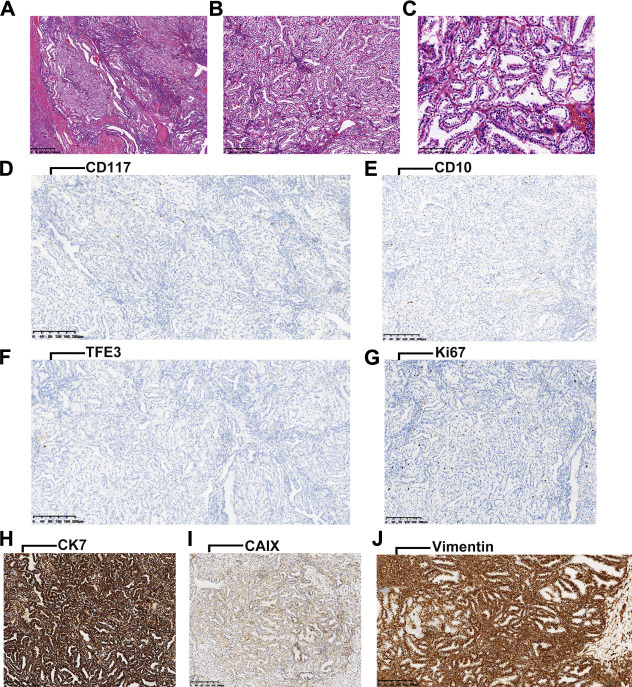
**(A-C)**, characteristic HE staining images of ccpRCC (arrangement of the nucleus is consistent with luminal polarity). **(D-G)**, negative for CD117, CD10, TFE3, and Ki67 in ccpRCC. **(H-J)**, positive for CK7, CAIX and Vimentin in ccpRCC.

The ccpRCCs exhibited a distinct papillary architecture and low nuclear grade. A histological feature of ccpRCC is the luminal polarity of the nucleus (Figures 1A–C). IHC analyses revealed that CD117, CD10, TFE3, and Ki67 (Figures 1D–G) were infrequently expressed in ccpRCC tissues that expressed readily detectable levels of CK7, CAIX, and vimentin (Figures 1H–J).

### Somatic Variants in Clear Cell Papillary Renal Cell Carcinoma

**FIGURE 2:**
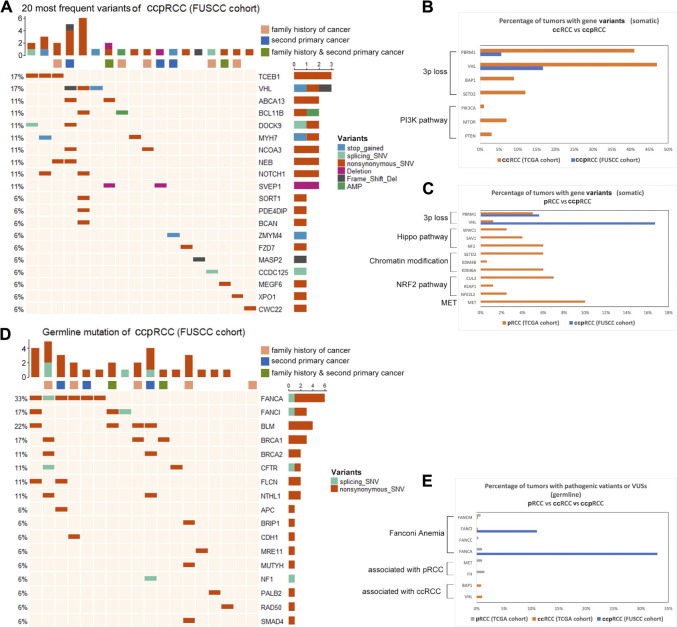
**(A)**, Top 20 somatic variants detected in ccpRCC (FUSCC cohort), various color represents corresponding various types of mutation as the figure shows. **(B)**, Comparison of somatic variants of ccpRCC and ccRCC (TCGA Cohorts). **(C)**, Comparison of somatic variants of ccpRCC and pRCC (TCGA Cohorts). **(D)**, Detected germline variants of ccpRCC. **(E)**, Comparison of germline variants of ccpRCC and ccRCC pRCC (TCGA cohort).

We identified 387 somatic variants in tissues of the 18 patients with ccpRCC. Detailed variants information was listed in Supplementary Table S1. And we compared the variants of ccpRCC with both ccRCC and pRCC from TCGA cohort. The 20 most frequently detected variants are displayed in Figure 2A. Among them, *TCEB1* (3/18) and *VHL* (3/18) were detected at the highest frequencies. In ccRCC, the most frequently mutated genes, which are associated with loss of chromosome 3p, encode components of the PI3K pathway. Only *VHL* (3p loss-associated) was mutated at a relatively high frequency in ccpRCC (Figure 2B). *MET* had the highest mutation frequency in pRCC, and the other most frequently mutated genes were associated with genes encoding the components of the Hippo pathway, chromatin modification, and the NRF2 pathway. In contrast, few of these variants were detected in ccpRCC tissues (Figure 2C). While the overall mutation rate for ccpRCC was found to be significantly less than either ccRCC (*p* < 0.0001) or pRCC (*p* < 0.0001), the detailed information of the overall mutation rate was listed in Supplementary Table S2. In summary, the overall somatic mutational characteristics of ccpRCC were distinct from those of ccRCC and pRCC.

### Germline Variants in Genes Encoding Components of the Fanconi Anemia Pathway (VAFAs) May Contribute to the Mechanism of Oncogenesis of Clear Cell Papillary Renal Cell Carcinoma

We detected germline variants in genes associated with Fanconi anemia in eight patients with ccpRCC (*FANCA*, 6/18; *FANCI*, 3/18) (Figure 2D). Furthermore, most of these patients (8/18) carried these VAFAs, among which five out of eight patients had second primary malignancy or family history of cancer. ccRCC and pRCC has very few VAFAs, while *FH*, *MET* (germline pathogenic variants of pRCC), *BAP1,* and *VHL* (germline pathogenic variants of ccRCC) were not detected in patients with ccpRCC (Figure 2E). The seven VAFA variants of uncertain significance were as follows (Table 4): *FANCA* c.2097A > G, *FANCA* c.3921G > C, *FANCA* c.3184G > A, *FANCA* c.3727G > A (potential pathogenic variants according to the prediction of PolyPhen-2). The *FANCA* mutation (c.2779–2A > T) is a known pathogenic variant. Germline VAFAs may be a molecular characterization of ccpRCC.

**TABLE 4:** Function prediction of the germline variants of genes associated with Fanconi anemia.

Gene	Genome variant	Classification	Variant type	ClinVar	PolyPhen-2 score	PolyPhen-2 prediction	Allele frequency in gnomAD
FANCA	c.2097A > G	VUS (potential pathogenic)	nonsynonymous SNV	uncertain significance	0.776	possibly damaging	NA
FANCA	c.3921G > C	VUS (potential pathogenic)	nonsynonymous SNV	uncertain significance	0.999	probably damaging	NA
FANCA	c.1328C > T	VUS	nonsynonymous SNV	uncertain significance	0.003	benign	6.57E-06
FANCA	c.4294G > T	VUS	nonsynonymous SNV	uncertain significance	0.214	benign	2.62764E-05
FANCA	c.3184G > A	VUS (potential pathogenic)	nonsynonymous SNV	uncertain significance	0.996	probably damaging	2.62757E-05
FANCI	c.3727G > A	VUS (potential pathogenic)	nonsynonymous SNV	uncertain significance	0.646	possibly damaging	NA
FANCI	c.1073C > G	VUS	nonsynonymous SNV	uncertain significance	0.002	benign	6.57488E-06
FANCA	c.2779–2 A > T	pathogenic	splicing SNV	—	—	—	NA
FANCI	c.3541–10T > C	intron	splicing SNV	—	—	—	NA

## Discussion

To our knowledge, the present study employed the largest sample size among studies that used WES to identify somatic variants in patients with ccpRCC. Furthermore, it is the first study to identify germline variants in such patients with ccpRCC. Here we identified 387 somatic variants in ccpRCC tissues of 18 patients. Moreover, our analyses reveal that the overall mutational characteristics of ccpRCC are distinct from those of ccRCC and pRCC. But *VHL* mutation frequency is also relatively high in ccpRCC, which implicates that this subtype may share some similarity with ccRCC. In ccpRCC patients, eight out of eighteen were found carrying VAFAs and 62.5% of the patients with VAFAs have second primary malignancy or family history of cancer. We discovered that germline VAFAs may play a key role in the oncogenesis of ccpRCC and may distinguish ccpRCC from ccRCC and pRCC.

Previous studies argue that *VHL* variants are undetectable in ccpRCC and that one criterion that precludes the diagnosis of ccpRCC is a *VHL* abnormality[22-25]. Conversely, a few studies claim that *VHL* variants may not distinguish ccpRCC from ccRCC. For example, a study of 15 tumors found that they are morphologically identical to ccpRCC and that most express CK7; however, molecular profiling indicates that a subgroup of these tumors carries VHL abnormalities [26]. Thus, this group is defined as “clear cell papillary-like RCC”[26]. Due to the rarity of this kind of renal carcinoma, previous studies did not find variants in common. Furthermore, histological analysis and immunophenotyping detected *VHL* variants among three patients diagnosed with ccpRCC. Similarly, we found here that three patients harbored a *VHL* mutation, although they did not exhibit symptoms of VHL syndrome. Furthermore, CK7 and CAIX exhibited diffuse IHC staining, whereas CD10 was undetectable. Thus, we conclude that *VHL* abnormalities may not distinguish ccpRCC from ccRCC.

A new subtype of kidney neoplasms, called “*TCEB1*-mutated RCC”, was reported in 2015[27]. Here we detected *TCEB1* variants in three patients with ccpRCC. However, the morphologies and immunophenotypes of the tumors reviewed by two experienced expert histopathologists suggested ccpRCC rather than *TCEB1*-mutated RCC. Thus, definitive differential diagnosis of these tumors requires further study. Furthermore, our comparisons of somatic variants of ccRCC and pRCC from TCGA data indicate that ccpRCC may be characterized by a low frequency of variants that contribute to the pathogenesis of ccRCC or pRCC. Thus, although the *VHL* mutation frequency was relatively high in ccpRCC, the overall mutational characteristics of ccpRCC were distinct from that of ccRCC and pRCC.

We are unaware of studies focused on the germline variants of ccpRCC. Interestingly, the high percentage of secondary primary malignancies and a family history of cancer were associated with the 18 patients with ccpRCC in this study. Thus, when we used targeted NGS to detect potential germline variants in our ccpRCC cohort, we found a high percentage of VAFAs. Furthermore, germline variants of *FH*, *MET* (associated with pRCC), and of *BAP1* and *VHL* (associated with ccRCC) were not detected in ccpRCC, indicating that the germline genotype of ccpRCC differs from those of ccRCC and pRCC.

In 1927, Guido Fanconi[28] treated three brothers suffering from aplastic anemia and was the first to describe the disease eponymously named “Fanconi anemia”. Studies of FA-associated genes and of the mechanism of FA indicate that VAFAs increase the risk of developing various cancers[29] because of defective DNA interstrand crosslink repair mediated by FANCs[30]. Here we identified nine germline VAFAs. In general, germline homozygous VAFAs are closely associated with FA, which increases the risk of developing hematological and non-hematological malignancies[31]. However, patients in our cohort did not show symptoms of FA, indicating that their VAFAs were heterozygous, which may increase the risk of developing ccpRCC. Moreover, although ccpRCC is generally considered an indolent neoplasm, the relatives of patients with ccpRCC are often diagnosed with diverse malignancies such as gastric cancer, breast cancer, and colorectal cancer. While there was no significant difference in the family history of cancer among ccpRCC patients with or without VAFAs. Furthermore, five patients in our present cohort were previously diagnosed with second primary malignancies. It is interesting to find that compared with ccRCC or pRCC, ccpRCC patients may be significantly correlated with a higher risk of developing second primary malignancy (5/18 versus 10–16% [19-21]). This may indicate that patients with ccpRCC should be more vigilant about developing second primary malignancy. These findings suggest that germline variants of VAFAs may be a molecular characterization of ccpRCC and may serve to distinguish ccpRCC from ccRCC and pRCC. This inference need to be validated in kinds of ways including larger population-based study and biological experiments and we have presented this as the main limitation of this research.

None of the patients in the present cohort experienced recurrence or metastasis, suggesting that surgery effectively treats ccpRCC. However, evidence indicates that recurrence and metastases cannot be excluded[10]. Thus, adjuvant chemotherapy should be considered. Although targeted therapy and immunotherapy are effective for treating ccRCC, they may be insufficient for treating ccpRCCs because of their phenotypic heterogeneity. The association of VAFAs with defective DNA repair and oncogenesis indicates that drugs that target proteins that mediate DNA repair, such as olaparib, may effectively treat ccpRCC.

Our research has certain limitations. Targeted NGS may limit the detection of germline variants, and our findings require verification through studies of a larger cohort. Since the VUSes are not evaluated according to standards in a clinical setting, it is hard to define the VAFAs as pathogenic mutations. Thus we just defined the VAFAs as a molecular characterization of ccpRCC. Furthermore, it is critically important to identify the underlying mechanism that generates the germline VAFAs associated with ccpRCC.

## Conclusion

In this study, we used NGS to explore potential somatic and germline variants in 18 ccpRCC patients. Molecular profiling of ccpRCC indicated that the overall somatic mutation characteristics of ccpRCC may be distinct from that of ccRCC and pRCC, and germline variants of VAFAs may be a molecular characterization of ccpRCC. Compared with ccRCC or pRCC, ccpRCC patients may be significantly correlated with a higher risk of developing second primary malignancy.

## Data Availability

Somatic mutation of ccRCC and pRCC from TCGA cohort were obtained from UCSC genome browser (https://xenabrowser.net/datapages/). Germline mutation of ccRCC and pRCC were obtained from supplementary materials of a previous study[14]. The data from the FUSCC cohort during the current study available from the corresponding author on reasonable request.

## Abbreviations

ccpRCC, clear cell papillary renal cell carcinoma; ccRCC, clear cell renal cell carcinoma; pRCC, papillary renal cell carcinoma; FA, fanconi anemia; TCGA, the cancer genome atlas; FUSCC, fudan university shanghai cancer center; VAFAs, variants associated with fanconi anemia pathway; WES, whole-exome sequencing; NGS, next generation of sequencing.

## References

[1] Sung H Ferlay J Siegel RL Laversanne M Soerjomataram I Jemal A Global Cancer Statistics 2020: GLOBOCAN Estimates of Incidence and Mortality Worldwide for 36 Cancers in 185 Countries. CA A Cancer J Clin (2021) 71(3):209–49. 10.3322/caac.21660 33538338

[2] Truong LD Shen SS. Immunohistochemical Diagnosis of Renal Neoplasms. Arch Pathol Lab Med (2011) 135(1):92–109. 10.1043/2010-0478-RAR.1 21204715

[3] Morlote DM Harada S Batista D Gordetsky J Rais-Bahrami S. Clear Cell Papillary Renal Cell Carcinoma: Molecular Profile and Virtual Karyotype. Hum Pathol (2019) 91:52–60. 10.1016/j.humpath.2019.05.011 31175917

[4] Gobbo S Eble JN Grignon DJ Martignoni G MacLennan GT Shah RB Clear Cell Papillary Renal Cell Carcinoma. Am J Surg Pathol (2008) 32(8):1239–45. 10.1097/pas.0b013e318164bcbb 18594469

[5] Aydin H Chen L Cheng L Vaziri S He H Ganapathi R Clear Cell Tubulopapillary Renal Cell Carcinoma: a Study of 36 Distinctive Low-Grade Epithelial Tumors of the Kidney. Am J Surg Pathol (2010) 34(11):1608–21. 10.1097/pas.0b013e3181f2ee0b 20924276

[6] Alshenawy HA. Immunohistochemical Panel for Differentiating Renal Cell Carcinoma with Clear and Papillary Features. Pathol Oncol Res (2015) 21(4):893–9. 10.1007/s12253-015-9898-7 25712789

[7] Rohan SM Xiao Y Liang Y Dudas ME Al-Ahmadie HA Fine SW Clear-Cell Papillary Renal Cell Carcinoma: Molecular and Immunohistochemical Analysis with Emphasis on the Von Hippel-Lindau Gene and Hypoxia-Inducible Factor Pathway-Related Proteins. Mod Pathol (2011) 24(9):1207–20. 10.1038/modpathol.2011.80 21602815

[8] Williamson SR. What Is the Malignant Potential of clear Cell Papillary Renal Cell Carcinoma?. Urol Oncol Semin Original Invest (2016) 34(9):420–1. 10.1016/j.urolonc.2016.05.035 27364705

[9] Diolombi ML Cheng L Argani P Epstein JI. Do Clear Cell Papillary Renal Cell Carcinomas Have Malignant Potential? Am J Surg Pathol (2015) 39(12):1621–34. 10.1097/pas.0000000000000513 26426379

[10] Gupta S Inwards CY Van Dyke DL Jimenez RE Cheville JC. Defining Clear Cell Papillary Renal Cell Carcinoma in Routine Clinical Practice. Histopathology (2020) 76(7):1093–5. 10.1111/his.14071 31989679

[11] Stratton MR Campbell PJ Futreal PA. The Cancer Genome. Nature (2009) 458(7239):719–24. 10.1038/nature07943 19360079 PMC2821689

[12] Lawrie CH Larrea E Larrinaga G Goicoechea I Arestin M Fernandez-Mercado M Targeted Next-Generation Sequencing and Non-Coding RNA Expression Analysis of clear Cell Papillary Renal Cell Carcinoma Suggests Distinct Pathological Mechanisms from Other Renal Tumour Subtypes. J Pathol (2014) 232(1):32–42. 10.1002/path.4296 24155122

[13] Mose LE Wilkerson MD Hayes DN Perou CM Parker JS. ABRA: Improved Coding Indel Detection via Assembly-Based Realignment. Bioinformatics (Oxford, England) (2014) 30(19):2813–5. 10.1093/bioinformatics/btu376 PMC4173014 24907369

[14] Huang KL Mashl RJ Wu Y Ritter DI Wang J Oh C Pathogenic Germline Variants in 10,389 Adult Cancers. Cell (2018) 173(2):355–e14. 10.1016/j.cell.2018.03.039 29625052 PMC5949147

[15] Gu Z Eils R Schlesner M. Complex Heatmaps Reveal Patterns and Correlations in Multidimensional Genomic Data. Bioinformatics (2016) 32(18):2847–9. 10.1093/bioinformatics/btw313 27207943

[16] The Cancer Genome Atlas Research Network. Comprehensive Molecular Characterization of Clear Cell Renal Cell Carcinoma*.* Nature (2013) 499(7456):43–9. 10.1038/nature12222 23792563 PMC3771322

[17] Linehan WM Spellman PT Ricketts CJ Creighton CJ Fei SS Davis C Comprehensive Molecular Characterization of Papillary Renal-Cell Carcinoma. N Engl J Med (2016) 374(2):135–45. 10.1056/nejmoa1505917 26536169 PMC4775252

[18] Clague J Lin J Cassidy A Matin S Tannir NM Tamboli P Family History and Risk of Renal Cell Carcinoma: Results from a Case-Control Study and Systematic Meta-Analysis. Cancer Epidemiol Biomarkers Prev (2009) 18(3):801–7. 10.1158/1055-9965.epi-08-0601 19240244 PMC2747496

[19] Chakraborty S Tarantolo SR Batra SK Hauke RJ. Incidence and Prognostic Significance of Second Primary Cancers in Renal Cell Carcinoma. Am J Clin Oncol (2013) 36(2):132–42. 10.1097/coc.0b013e3182438ddf 22441339 PMC3383896

[20] Joung JY Kwon W-A Lim J Oh C-M Jung K-W Kim SH Second Primary Cancer Risk Among Kidney Cancer Patients in Korea: A Population-Based Cohort Study. Cancer Res Treat (2018) 50(1):293–301. 10.4143/crt.2016.543 28421722 PMC5784635

[21] Rabbani F Reuter VE Katz J Russo P. Second Primary Malignancies Associated with Renal Cell Carcinoma: Influence of Histologic Type. Urology (2000) 56(3):399–403. 10.1016/s0090-4295(00)00682-8 10962302

[22] Wolfe A Dobin SM Grossmann P Michal M Donner LR. Clonal Trisomies 7,10 and 12, normal 3p and Absence of VHL Gene Mutation in a Clear Cell Tubulopapillary Carcinoma of the Kidney. Virchows Arch (2011) 459(4):457–63. 10.1007/s00428-011-1137-3 21822960

[23] Favazza L Chitale DA Barod R Rogers CG Kalyana-Sundaram S Palanisamy N Renal Cell Tumors with Clear Cell Histology and Intact VHL and Chromosome 3p: A Histological Review of Tumors from the Cancer Genome Atlas Database. Mod Pathol (2017) 30(11):1603–12. 10.1038/modpathol.2017.72 28731045

[24] Hes O Compérat EM Rioux-Leclercq N. Clear Cell Papillary Renal Cell Carcinoma, Renal Angiomyoadenomatous Tumor, and Renal Cell Carcinoma with Leiomyomatous Stroma Relationship of 3 Types of Renal Tumors: A Review. Ann Diagn Pathol (2016) 21:59–64. 10.1016/j.anndiagpath.2015.11.003 26897641

[25] Inoue T Matsuura K Yoshimoto T Nguyen LT Tsukamoto Y Nakada C Genomic Profiling of Renal Cell Carcinoma in Patients with End-Stage Renal Disease. Cancer Sci (2012) 103(3):569–76. 10.1111/j.1349-7006.2011.02176.x 22145865 PMC7713630

[26] Gonzalez ML Alaghehbandan R Pivovarcikova K Michalova K Rogala J Martinek P Reactivity of CK7 Across the Spectrum of Renal Cell Carcinomas with Clear Cells. Histopathology (2019) 74(4):608–17. 10.1111/his.13791 30444288

[27] Hakimi AA Tickoo SK Jacobsen A Sarungbam J Sfakianos JP Sato Y TCEB1-Mutated Renal Cell Carcinoma: A Distinct Genomic and Morphological Subtype. Mod Pathol (2015) 28(6):845–53. 10.1038/modpathol.2015.6 25676555 PMC4449825

[28] Fanconi G. Familiare Infantile Perniziosaartige Anamie (pernizioses Blutbild und Konstitution). Jahrbuch Kinderheilk (1927) 117:257–80.

[29] Osorio A Bogliolo M Fernández V Barroso A de la Hoya M Caldés T Evaluation of Rare Variants in the New Fanconi Anemia GeneERCC4(FANCQ) as Familial Breast/Ovarian Cancer Susceptibility Alleles. Hum Mutat (2013) 34(12):1615–8. 10.1002/humu.22438 24027083

[30] Kim H D'Andrea AD. Regulation of DNA Cross-Link Repair by the Fanconi Anemia/BRCA Pathway. Genes Develop (2012) 26(13):1393–408. 10.1101/gad.195248.112 22751496 PMC3403008

[31] Stecklein SR Jensen RA. Identifying and Exploiting Defects in the Fanconi Anemia/BRCA Pathway in Oncology. Translational Res (2012) 160(3):178–97. 10.1016/j.trsl.2012.01.022 22683426

